# A Systematic Review of the Prevalence and Risk Factors of Depression among Iranian Adolescents

**DOI:** 10.5539/gjhs.v5n3p16

**Published:** 2013-01-15

**Authors:** Homeira Sajjadi, Seyed Hossein Mohaqeqi Kamal, Hassan Rafiey, Meroe Vameghi, Ameneh Setareh Forouzan, Masoomeh Rezaei

**Affiliations:** 1 Social Determinant of Health Research Center, University of Social Welfare and Rehabilitation Sciences, Tehran, Iran; 2 Department of Social Welfare Management, University of Social Welfare and Rehabilitation Sciences, Tehran, Iran; 3 Department of Occupational Therapy, University of Social Welfare and Rehabilitation Sciences, Tehran, Iran

**Keywords:** depression, prevalence, risk factor, adolescent, systematic review, Iran

## Abstract

Depression is the most common mood and psychiatric disorder. The aim of this comprehensive study was to provide a complete picture of the prevalence and risk factors of depression. The study employed a systematic review methodology, searching Iranian and international databases. After screening and evaluating the articles, a synthesis of 53 articles was accumulated. A meta-analysis of the studies showed that the prevalence of children and adolescent depression was 43.55% using the BDI, 15.87 % using SCL-90, and 13.05% using CDI. Also, the prevalence of depression was higher among girls than boys based on the BDI and CDI results. The most important factors contributing to depression were: the female sex, poor inter-parental relationship, poor adolescent-parent relationship, low socio-economic status (SES), state of parenting styles, low level of parental education, and poor academic performance. The comparatively high prevalence of depression among Iranian adolescents call for further investigation and measures.

## 1. Introduction

Depression is a common psychiatric disorder in all regions of the world (Costello, 2006). It is predicted that depressive disorders will become the first leading cause of disease burden by 2015 (WHO, 2008). Similarly, depressive disorders are also a common mental health problem among adolescents worldwide (Lopez, 2006), with the lifetime prevalence of 20–25% (Allen et al., 2007; Murphy, 2000). Prevalence estimates vary widely across studies and countries (Lopez, 2006; Fleitlich, 2004; Pillai, 2008). In the same vein, the prevalence of depression among adolescents in Iran is variable between 14.77% (Janboozorgi, 2005) to 72% (Monirpour, 2004).

Moreover, depression among adolescents is more often missed than it is in adults (Leaf, 1996), possibly because of the prominence of irritability, mood reactivity, and fluctuating symptoms. In some cases, depression among adolescents can be viewed as an early onset and sub-form of the equivalent adult disorder owing to its strong links with recurrence later in life (Birmaher, 2004). The prevalence of depression among children is low (<1% in most studies) (Kessler, 2001) with no gender differences, and then seems to rise substantially throughout adolescence (Green, 2005). Many factors could explain the documented post-pubertal rise in depression prevalence as adolescence is a developmental period characterized by pronounced biological and social changes (Patton, 2007; Cyranowski, 2000).

It is important to identify the risk factors associated with depression among adolescents, as this can help control and prevent depression. Many risk factors have been reported in the literature such as the female sex (Danesh, 2007; Norozi, 2006; Genaabadi, 2010; Rahmani, 2007), poor inter-parental relationship (Ghorbani, 2003; Zahirodin, 2005; Kooroshnia, 2007), low socioeconomic status (Rostamzadeh, 2001; Shojaeezadeh, 1999; Modabernia, 2005), low level of parents’ education (Mirzaand & Jenkins, 2004; Shahnazi, 2007), and poverty (Mirza & Jenkins, 2004). Given its vast personal, social, and economic impacts, depressive disorders create significant demands on individuals, health service providers, and the society as a whole (NICE, 2009; Thomas & Morris, 2003). Furthermore, to assist policy development for tackling adolescent depression, empirical investigation and evidence are the prerequisites. Thus, considering the importance of depression among Iranian adolescents, a systematic review of the prevalence and risk factors of depression among adolescent in Iranian studies was conducted as no such work has been carried out to the best of our knowledge. The main question posed in the current study was as follows: (a) what is the mean prevalence of depressive disorders in adolescence in Iran; and (b) what are the associated factors?

## 2. Methods

### 2.1 Search Strategy

Quantitative studies were searched on and downloaded from Medline (pub med), SID (Scientific Information Database, www.sid.ir), Irandoc (Iranian Research Institute for information Science and Technology, Irandoc.ac.ir), Iranmedex (www.iranmedex.com), Magiran (www.magiran.com) and Iranpsych (Iranian Databases on Mental Health, Psychiatric and Psychological Research, http://iranpsych.tums.ac.ir). The electronic search was performed with no specification of language including articles from 1997 to 1 march 2011. The five keywords included, “*Depression, Dysthymia, Melancholia, Mood disorder*, and *Iran*”. In this case, Boolean operators were used. References of the downloaded papers and previous reviews were critically studied through to find any relevant study missed by the electronic search. Unpublished studies/gray literature were not searched in this review.

### 2.2 Inclusion/Exclusion Criteria

A structured checklist was designed to record the adequacy of the retrieved papers from the electronic search. All papers were initially screened by reviewing the titles. In so doing, two reviewers individually evaluated each study to decide on its inclusion or exclusion in the systemic review. Reliability and validity were exactly checked in process of reviewing. In the case of any disagreement or vagueness; the three researchers discussed and reached a consensus. Where doubt remained, the paper was studied in its entirety. For inclusion, the articles had to (a) use an Iranian sample, (b) be quantitative, (c) deal with depression prevalence and risk factors, (d) include schoolchildren aged 7 to 18, (f) be conducted over the past 15 years.

### 2.3 Quality Assessment

The research team developed two checklists for evaluating the quality of descriptive and analytical studies. After reviewing the relevant instruments, such as the *Critical Appraisal Skills Program* (Public Health Resource Unit, 2006), a new checklist, suitable for our purposes, was developed. The reliability of the checklist was assessed in a pilot phase before being applied to the selected studies. The methodological checklist for descriptive studies and the descriptive findings of the analytical studies assessed the following criteria: (a) sample size, (b) sampling method, and (c) reliability and validity of data collection. The checklist for analytical studies covered the following areas: (a) appropriateness of the aim of research with the study design, (b) controlling of intervening variables, (c) correct selection of samples in the study group(s), and (d) correct measurements and appropriateness of statistical analyses (e.g. the odds ratio, correlation coefficients, *p* values and confidence interval).

### 2.4 Data Extraction

A structured checklist was used for the extraction of information on the year of publication, study design, population, sample size, prevalence, depression degree, risk factors, inclusion/exclusion criteria (including age, gender, and education), and instruments. Additional information on study results was extracted with respect to the type of instruments.

### 2.5 Statistical Analysis

A narrative synthesis of the extracted studies was formed to address the questions of the review. For the meta-analysis, after exclusion of irrelevant data, the size of the sample leading to each finding was considered and ascribed a ‘weight’, where-after, weight averages were calculated if the findings were sufficiently homogenous. In the case of significant heterogeneity, the findings were grouped into subcategories, a well-known method for approaching the heterogeneity of data (Khan et al., 2003).

## 3. Results

**Figure 1:**
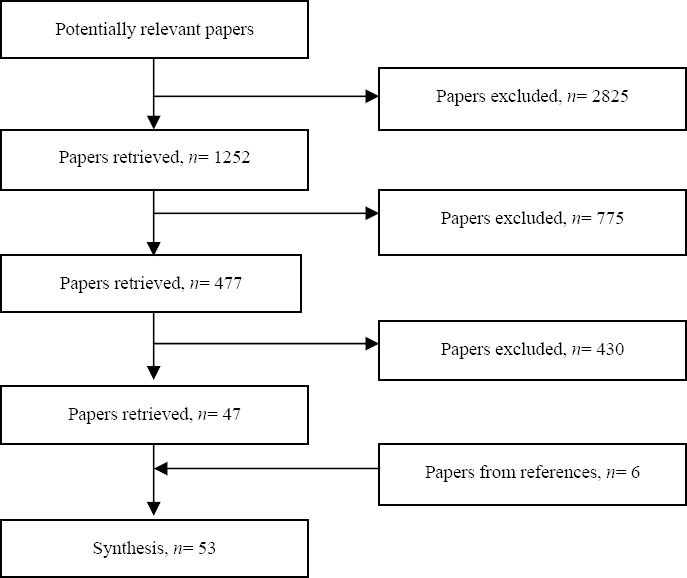
The process of screening article for systematic review

The electronic search yielded 4,077 results, but an initial review of titles led to the exclusion of 2,825 as they were either duplicated or published before the studied period (i.e. 1997-2011). The abstracts of the remaining 1,252 articles were reviewed to identify potentially relevant papers. Consequently, 775 were excluded mainly Due to irrelevance or because of their different study design from this review. The full texts of the remaining 477 articles were reviewed and a further 430 put aside as they had not studied adolescent groups. Thus 47 articles from which data related to adolescents were extracted and used in our review. Also, the *References* sections of the remaining papers and previous reviews were hand-searched and six additional studies were identified as relevant. Finally, a synthesis of 53 quantitative studies was considered qualified after the review process, an illustration of which is provided Figure 1.

Results are presented under two sections namely prevalence (31 articles) and risk factors (40 articles). It should be mentioned that some papers contained data on both prevalence and risk factors, thus, included in the both categories.

### 3.1 Prevalence of Depression among Adolescents

In total, 31 studies were assigned to the prevalence of depression among Iranian adolescents. Key features of the papers are summarized in Table 1. Twelve article shad been performed using the Beck Depression Inventory (BDI) (Beck, 1961), seven articles using the Symptoms Checklist-90 (SCL-90) (Derogatis et al., 1973), five articles using the Children Depression Inventory (CDI) (Kovacs, 1981), two articles using the Zung Questionnaire (Zung, 1965), two articles using the General Health Questionnaire (GHQ) (Goldberg & Williams, 1988), one article using the Rutter Questionnaire (Rutter, 1990), one article using the Kiddie Schedule for Affective Disorders and Schizophrenia (K-SADS) (Endicott & Spitzer, 1978), one article using the Depression Scale for Children (CES-DC) (Kovacs, 1981), and one article a clinical interview. In one study (Janboozorgi, 2005); the BDI and CDI instruments were used together.

**Table 1:** Key features of papers on prevalence

Author	Population	Sample size	Instrument	Prevalence
Rostamzadeh et al. (2007) 14-18 Y/O	Oromianhigh school girls	3,023	BDI	68.1 (T)
Zargham-Borojeni et al. (2002) >=18 Y/O	Isfahani adolescents	323	BDI	43.4 (T), 55.5 (G), 30.8 (B)
Monirpour et al. (2005) 14-18 Y/O	Sari students	377	BDI	72 (T), 75.3 (G), 65.7 (B)
Zahirodin et al. (2005) 14-18 Y/O	Firozkohi high school students	604	BDI	65.35 (T), 73 (G), 58 (B)
Chelsi-Roshan et al. (2003) 14-18 Y/O	Ardebilihigh school students	967	BDI	26 (T)
Rahimi-Kian et al. (2004) 14-18 Y/O	Karajihigh school students	264	BDI	68.4 (T)
Soki et al. (2007) 14-18 Y/O	Kashanihigh school girls	762	BDI	53.3 (T)
Narimani et al. (2000) 14-18 Y/O	Ardebilihigh school girls	127	BDI	43 (T)
Johari et al. (1999) 17-18 Y/O	Ilamihigh school students	372	BDI	29.10 (T),31 (G), 27 (B)
Mogharab et al. (2009) 14-18 Y/O	Birjandihigh school students	450	BDI	58.8 (T)
Janboozorgi et al. (2005) 12-18 Y/O	Tehrani secondaryand high school students	1,551	BDI	14.77 (T), 16.14 (G), 13.4 (B)
Modabernia et al. (2005) 14-18 Y/O	Rashtihigh school students	4,020	BDI	34 (T)
Zarabi et al. (2001) 14-18 Y/O	Rashtihigh school students	557	SCL-90	16.2 (T)
Hosseini et al. (2002) 14-18 Y/O	Sarihigh school students	1,536	SCL-90	0.88 (T),1.04 (G), 0.74 (B)
Gena-Abadi et al. (2010) 14-18 Y/O	Saravanihigh school students	125	SCL-90	26.06 (T),20.85 (G), 14 (B)
Sepehrmanesh et al. (2002) 14-18 Y/O	Kashani high schoolstudents	400	SCL-90	1.04 (T), 1.22 (G), 0.97 (B)
Hosseinifard et al. (2000) 14-18 Y/O	Rafsanjani high schoolstudents	830	SCL-90	2. 4 (T),3.1 (G), 1.6 (B)
Masoodzadeh et al. (2002) 14-18 Y/O	Sarihigh school students	1,068	SCL-90	0.60 (T),0.62 (G), 0.58 (B)
Hosseini et al. (1999) 14-15 Y/O	Sarisecondary school students	350	SCL-90	1.02 (T), 1.41 (G), 0.7 (B)
Ostovar et al. (2003) 12-18 Y/O	Shirazi secondaryand high school students	403	CDI	15 (T), 17.07 (G), 11.59 (B)
Rajabi et al. (2001) 14-16 Y/O	Ahvazi secondaryand high school students	400	CDI	25.6 (T), 25.5 (G), 25.1 (B)
Ghorbani et al. (2003) 8-16 Y/O	Isfahani adolescents	117	CDI	8 (T)
Abdolahian et al. (1998) 10-12 Y/O	Mashhadiadolescents	2,071	CDI	25.6 (T), 25.5 (G), 25.1 (B)
Janboozorgi et al. (2005) N=18 Y/O	Tehranielemenntary and high school students	1551	CDI	10.3 (T),13.1 (G), 7.6 (B)
Motaghipour et al. (2005) 12-18 Y/O	Isfahani adolescents	346	GHQ	1 (T),1.2 (G), 0.6 (B)
Sadeghian et al. (2008) 14-18 Y/O	Hamadani high school girls	600	GHQ	45.8 (T)
Habibpour et al. (2001) 14-18 Y/O	Isfahanhigh school students	400	ZONG	29.5 (T)
Shojaeezadeh (1999) 14-18 Y/O	Kazeronihigh school students	240	ZONG	52.9 (T)
Shahnazi et al. (2007) 12-18 Y/O	Tabrizi adolescents	364	CES-DC	75.82 (T)
Ranjbar et al. (1998) >=16 Y/O	Tabrizi adolescents	252	Rutter	55.5 (G), 44.5 (B)
Aahmad-Khaniha et al. (2001) >=18 Y/O	Tehranistreet children	87	K-SADS	61.4 (T), 86.7 (G), 48.2 (B)
Sayari et al. (1998) 14-18 Y/O	Tehrani high school students	388	Clinical interview	72 (T), 75.3 (G), 65.7 (B)

*Note*. T = total prevalence, G = prevalence among girls, B = prevalence among boys

As the current study could only the papers using the same instruments, and thereby obtain the weighted average, the mean prevalence of depression was based on three instruments including the BDI, Scl-90 and CDI. The 12 articles employing the BDI had the weighted average of 55.43% (*N*=12,851). The weighted average five of the seven articles using Scl-90 was 15.87% (*N* = 2,262) (The two others Masoodzadeh’s (2002) and Hosseini’s (1999) articles were excluded due to the lack of estimates of prevalence. The weighted average of the five articles using CDI was calculated to be 13.05% (*N*=4542).

### 3.1.1 The Prevalence of Depression by Gender

Only the studies using the BDI and CDI as instruments furnished information on the prevalence of depression among boys and girls as two distinct groups. Thereby, the prevalence of depression by gender is presented on the basis of these two instruments. Out of the five articles measuring the prevalence of depression with [the] CDI, four articles provided the mean estimate, although Ghorbani’s (2003) article was omitted due to lacking any estimate by gender. Subsequently, the weighted average of the four studies showed that the prevalence of depression was 15.32% among girls (*n*= 2,155) and 11.05% among boys (*n*= 2,270).

Eight articles which have utilized the BDI, provided prevalence mean estimate were included. The weighted average of the eight studies showed that the prevalence of depression was 57.69% among girls (*n*=5,498) and 30.09% among boys (*n*=1,652). Although the utilization of these instruments yielded diverse prevalence estimates of depression by gender, in both cases the mean prevalence of depression was higher for girls than boys.

### 3.2 Risk Factors associated with Depression among Adolescents

Of the 31 articles related to prevalence of depression among adolescents, 18 papers also presented information on the factors associated with adolescent depression. In addition, 22 articles specifically referred to these factors. Thus, a total of 40 articles were considered in this section, the key features of which are summarized in Table 2.

**Table 2:** Risk factors associated with adolescent depression

Author	Factors associated with adolescent depression												
	SES	PR	IA	RAP	SE	P	AS	ME	FE	PS	Age	L	S&D
Shojaeezadeh (1999)	*	*						*					
Hosseini et al. (2002)											*		
Gena-Abadi et al. (2010)												*	
Sepehrmanesh et al. (2002)													*
Motaghipour et al. (2005)								*					
Janboozorgi et al. (2005)													
Modabernia et al. (2005)	*												
Ghorbani et al. (2003)		*						*	*				
Abdolahian et al. (1998)	*				*							*	
Shahnazi et al. (2007)				*	*		*	*	*				
Aahmad-Khaniha et al. (2001)	*												
Monirpour et al. (2005)	*								*		*		
Zahirodin et al. (2005)		*		*			*			*			*
Chelsi-Roshan et al. (2003)													
Soki et al. (2007)				*					*				
Narimani et al. (2000)													
Johari et al. (1999)													
Mogharab et al. (2009)													
Ghafari et al. (2005)													
Kaheni et al. (2000)		*											
Kooroshnia et al. (2007)		*											
Khoshnevisan et al (2011)							*						
Norozi et al. (2006)		*											
Danesh et al. (2007)										*			
Sadeghzadeh et al. (2010)		*								*			
Rahmani et al. (2007)										*			
Navabakhsh et al. (2000)						*							
Yosefi et al. (2000)						*							
Tahmasian et al. (2007)							*						
Neshatdust et al. (2005)		*		*				*					
Borojeni et al. (2001)				*						*			
Khanjani et al. (2010)													
Pourshirazi et al. (2007)			*										
Rostamzadeh et al. (2001)	*	*		*				*	*		*	*	
Fakhari et al. (2001)				*									
Seyed-Amini et al. (2009)													
Rahimi-Kian et al. (2001)											*		
Abdi et al. (2009)						*	*						
Seify-Gandomani et al. (2009)										*			
Peyvastegar et al. (2008)						*							

Author	Factors associated with adolescent depression												
	TS	D	Field	Gender	MJ	FJ	PD	O&O	SC	NH	HD	EA	V&P
Shojaeezadeh (1999)													
Hosseini et al. (2002)				*									
Gena-Abadi et al. (2010)				*									
Sepehrmanesh et al. (2002)				*									
Motaghipour et al. (2005)				*									
Janboozorgi et al. (2005)	*	*		*									
Modabernia et al. (2005)	*		*	*									
Ghorbani et al. (2003)					*	*	*						
Abdolahian et al. (1998)				*			*		*	*	*		
Shahnazi et al. (2007)		*				*						*	*
Aahmad-Khaniha et al. (2001)				*		*							
Monirpour et al. (2005)			*	*									
Zahirodin et al. (2005)				*		*				*			*
Chelsi-Roshan et al. (2003)	*	*											
Soki et al. (2007)													
Narimani et al. (2000)	*												
Johari et al. (1999)				*						*			
Mogharab et al. (2009)	*												
Ghafari et al. (2005)											*		
Kaheni et al. (2000)				*									
Kooroshnia et al. (2007)													
Khoshnevisan et al. (2011)													
Norozi et al. (2006)				*									
Danesh et al. (2007)				*									
Sadeghzadeh et al. (2010)													
Rahmani et al. (2007)				*									
Navabakhsh et al. (2000)													
Yosefi et al. (2000)													
Tahmasian et al. (2007)													
Neshatdust et al. (2005)						*							*
Borojeni et al. (2001)													
Khanjani et al. (2010)											*		
Pourshirazi et al. (2007)													
Rostamzadeh et al. (2001)					*	*							
Fakhari et al. (2001)													
Seyed-Amini et al. (2009)								*					
Rahimi-Kian et al. (2001)		*								*			
Abdi et al. (2009)													
Seify-Gandomani et al. (2009)											*		
Peyvastegar et al. (2008)													

**Note:** IPR =inter-parental relationship, RAP =relationship between adolescent-parents, ME = mother's education, FE = father's education, PS = parenting styles, AS = academic success, SES = socioeconomic status, P = personality, L = location, S&D = smoking and drug abuse, TS = type of school, D = degree, IA= internet addiction, MJ = mother's job, FJ = father's job, PD = parents’ divorce, O&O = overweight and obesity, SC = school change, NH = number of households, HD = history of mental illness, EA = extracurricular activities, SE = stressful events, V&P = violence and punishment.

As can be inferred, more than 26 factors were associated with adolescent depression including the being female sex (15 articles), poor inter-parental relationship (nine articles), poor adolescent-parent relationship (seven articles), low socioeconomic status (six articles), low level of the mother’s education (six articles), authoritarian parenting styles (six articles), low level of the father’s education (five articles) and poor academic performance (five articles). All factors had a positive relationship with adolescent depression, except the last one.

Given the diversity of risk factors and lack of common data, there was no possibility for a meta-analysis. Therefore, the studies were only divided into subgroups and reported. These factors can be placed in five main subgroups:

1) Demographic factors including age, gender, parents’ education, socioeconomic status, location, and number of households.
2) Communicational problems including parents’ divorce, relationship between adolescent-parents, inter-parental relationship, parenting styles, violence and punishment.
3) Educational factors including poor academic performance, school change, extracurricular activities, type of school, degree, and field of education.
4) Psychological factors including a history of mental illnesses in the family, stressful events (such as death and divorce), and personality.
5) Other factors including overweight and obesity, internet addiction, smoking and drug abuse.

## 4. Discussion

In measuring depression prevalence among Iranian adolescents three instruments were used including the BDI, SCL-90 and CDI. Accordingly, the prevalence mean using the BDI was 43.55%, varying between 14.77% (Janboozorgi, 2005) to 72% (Monirpour, 2005). The mean prevalence using the SCL-90 was 15.87%, varying between 0.60% (Masoodzadeh, 2002) to 41.1% (Hosseini, 1999). In addition, the prevalence with CDI was 13.05%, ranging between 8% (Ghorbani, 2003) to 25.6% (Rajabi, 2001).

Some of research found an increased prevalence of depression among adolescents (El-Missiry, 2012; Sarkar, 2012; Fan, 2011; Rahman, 2009). El-Missiry (2012) showed that depression in a sample of Egyptian secondary school female students was estimated to be 15.3% using the CDI (El-Missiry, 2012). Sarkar (2012) found that the prevalence of depressive disorders in school children of suburban India was 3.13% using K-SADS-PL (Sarkar, 2012). Fan (2011) showed that the prevalence of children depression in Shanghaian children aged 8-12 years was 1.60% with K-SADS (Fan, 2011). Furthermore, the prevalence of depressive disorders among young women (16-18 years old) in rural Pakistan was 4.4% with SCID (Rahman, 2009).

It seems evident that the prevalence of adolescent depression in Iranian studies was higher from other countries. The reason for this difference May be attributed due to diverse geographical environments, economic and cultural characteristics, and etiological and diagnostic instruments. However, population prevalence estimates may also vary widely across studies and countries due to methodological differences (from 1.6% in China (Fan, 2011) to 15.3% in Egypt (El-Missiry, 2012).

The prevalence of depression among boys and girls was based on the BDI and CDI as instruments. Accordingly, the prevalence of depression among male adolescents based on CDI was 11.05% and among female adolescents 15.32%. In addition, the prevalence of depression among boys was also 30.09% and among girls 57.69% based on the BDI. Although the instruments reported different prevalence estimates, the mean prevalence of depression was higher among girls than boys.

One of the main epidemiological findings is the emergence of a strong female predisposition toward depression after puberty (Hyde, 2008). Although the reasons for this post-pubertal onset are not fully understood, adolescent depression seems more closely tied to female hormonal changes than to age (Angold, 1999). However, hormonal changes alone rarely produce the behavioral or neural signs of depression (Soares, 2008) and are more likely to contribute by sensitizing the brain to the adverse effects of stress (Hyde, 2008; Angold, 1999; Goodyer, 2000). Thus, the post pubertal gender difference in depression might in part result from increased exposure to stressors and hormonally. Finally, although depression is generally more common among girls, recognizing its extent among boys is also important. Moreover, in some subgroups (e.g., patients with Neurodevelopmental and medical disorders) this gender difference might be greatly weakened, absent, or even reversed e.g. social phobia, attention deficit hyperactivity disorder, and alcohol and drug misconduct (van Noorden, 2010).

Results about factors associated with depression showed that more than 26 factors were associated with adolescent depression. The female sex, poor inter-parental relationship, poor adolescent-parents relationship, low socioeconomic status, low education of the mother, authoritarian parenting styles, low education of the father and poor academic performance were factors most associated with adolescent depression.

Furthermore, it seems that the gender differences cause differences in depression *prevalence*. Teenage girls are more prone to have mood disorders and depression such that the incidence of depression among girls compared to boys is almost two to one. These differences are probably affected by multiple factors such as biological, psychological, social, and cognitive ones (Oldehinkel, 1998).

Various researchers have found similar results as explicated here, that is, the prevalence of depression among women in Pakistan has been associated with poverty, lack of education, relationship problems, and social status of women (Mirza & Jenkins, 2004). In addition, El-Missiry (2012) in a sample of Egyptian secondary school female students showed that depression was associated with poor academic performance, quarrelsome family atmosphere, socioeconomic status, unfavorable life events, and a family history of psychiatric disorders.

Lack of mutual understanding and agreement between parents may foster a feeling of inferiority and insecurity among children. In addition, if quarrels happen very often, anxiety and depression will probably become permanent and irritating for children. Considerable research has documented that prolonged exposure to inter parental conflict (IPC) has negative consequences for children and adolescents, including increased risk for externalizing and internalizing problems (e.g., Davies & Windle, 2001; Emery, 1988).

Family structure and especially parent-adolescent relationship may affect adolescent depression (Puskar, 1999). To prevent depression, one of the optimal parenting styles is a democratic approach. This includes distributing affection and attention evenly among one’s children, not imposing undue influence and control upon them, and leaving them free to make their own decisions. Field et al.’s (2001) study on 29 high school adolescents in the United States shows that teens with high depression degrees had a poor relationship with their parents.

Economic hardships may trigger negative parent-children interactions, leading to more intense feelings of depression, isolation, and loneliness among children (Lemperes, 1997). According to Morgan (as cited in Shahnazi, 2008), poverty and race may put children and adolescents at risk of depression.

Nrugham et al. (2008) findings found that the prevalence of depression was higher in teenagers whose parents had lower levels of education compared to those whose parents had received university or higher education. a large body of literature that highlights the importance of parental education and income for determining children’s behaviors (Carlson & Corcoran, 2001; Duncan & Brooks-Gunn, 2000), educational performance (Magnuson, 2007), and health (Bloom, Cohen, & Freeman, 2009).

Moreover, longitudinal investigations have demonstrated that adolescents who reported sub-syndromal levels of depressive symptoms (i.e., symptoms below the threshold of diagnosis) were at an increased risk of major depressive episodes well into adulthood, culminating in anxiety disorders, nicotine or alcohol addiction or both, educational underachievement, unemployment, early parenthood, and suicide attempts (Burns et al., 2004; Cuijpers, Graaf, & van Dorsselaer, 2004; Fergusson, Horwood, Ridder, & Beautrais, 2005; Lewinsohn, Solomon, Seeley, & Zeiss, 2000).

## 4. Conclusion

It seems that there is a high prevalence of depression in adolescents. Depression in Iranian adolescents is very important, especially for girls. The most important factors contributing to depression were: the female sex, poor inter-parental relationship, poor adolescent-parent relationship, low socio-economic status (SES), state of parenting styles, low level of parental education, and poor academic performance. Also, despite the global importance of depression in adolescence and a considerable and growing body of literature, still many areas merit attention. Further development of pragmatic, cost-effective methods of detecting, measuring, and treating adolescent depression in non-specialist contexts and low-income and middle-income countries is an important priority in view of the scarcity of resources. Lack of research on preventing relapse is also noticeable. Finally, prevention strategies seem important because of the complexities and costs associated with treatment of depression among adolescents. Furthermore, enhanced coping strategies for depression among children and adolescents and review and reform of the educational system are the most important factors in prevention of depression in later life.
